# The influence of *in vitro* gastrointestinal digestion and fecal fermentation on the flowers of *Juglans regia*: Changes in the active compounds and bioactivities

**DOI:** 10.3389/fnut.2022.1014085

**Published:** 2022-09-07

**Authors:** Ximeng Jin, Yuerong Ru, Xuechun Zhang, Huan Kan, Ping Xiang, Xuemei He, Jian Sun, Xiahong He, Zhengxing Wang

**Affiliations:** ^1^Key Laboratory for Forest Resources Conservation and Utilization in the Southwest Mountains of China, Ministry of Education, Southwest Forestry University, Kunming, China; ^2^College of Life Science, Southwest Forestry University, Kunming, China; ^3^Institute of Environmental Remediation and Human Health, Southwest Forestry University, Kunming, China; ^4^Guangxi Key Laboratory of Fruits and Vegetables Storage-Processing Technology, Guangxi Academy of Agricultural Sciences, Nanning, China; ^5^College of Horticulture and Landscape, Southwest Forestry University, Kunming, China

**Keywords:** flowers of *Juglans regia*, *in vitro* gastrointestinal digestion, fecal fermentation, polyphenols, short-chain fatty acids, gut microbiota

## Abstract

The objective of the research was to investigate the digestion and fecal fermentation characteristics of the flowers of *Juglans regia* (FJR), by using *in vitro* simulated digestion model (oral, gastric, and intestine) as well as colonic fermentation. As a result, the contents of most active substances and functional activities of FJR were decreased as the digestion proceeded, and showed a trend of first increasing and then decreasing in the fecal fermentation phase. In the oral digestion phase, the total phenolic and total flavonoid contents were released most with the values of 11.43 and 9.41 μg/mg, respectively. While in the gastric digestion phase, the antioxidant abilities, α-glucosidase and α-amylase inhibitory abilities were the weakest. By using high-performance liquid chromatography, 13 phenolic acids and 3 flavonoids were detected. Of these, the highest number of identified compounds were found in the undigested and the oral digestion stages, which were mainly salicylic acid, epicatechin, 3,5-dihydroxybenoic acid, vanillic acid, and protocatechuic acid. However, great losses were observed during the gastric and intestinal digestion stages, only epicatechin, salicylic acid, and protocatechuic acid were found. Surprisingly, fecal fermentation released more abundant phenolic substances compared to gastric and intestinal digestion. Additionally, FJR reduced the pH values in the colonic fermentation system, significantly promoted the production of short-chain fatty acids, and regulated the microbe community structure by improving the community richness of beneficial microbiota. This indicated that FJR had the benefit to improve the microorganismal environment in the intestine. Further Kyoto Encyclopedia of Genes and Genomes pathway analysis revealed that FJR could suppress the metabolic pathways related to diseases, such as infectious diseases, metabolic diseases and neurodegenerative diseases. In conclusion, although the bioactivities of FJR decreased significantly after *in vitro* gastrointestinal digestion and fecal fermentation, it still maintained certain antioxidant and hypoglycemic ability *in vitro*. This study described the detailed changes in the active compounds and bioactivities of FJR during *in vitro* gastrointestinal digestion and fecal fermentation, and its effects on microbiota composition and SCFAs levels in feces. Our results revealed the potential health benefits of FJR, and could provide a reference for its further research and development.

## Introduction

The flower of *Juglans regia* (FJR) is one of the primary by-products of walnuts production (1). Although walnuts have been reported to have multifaceted functions including anti-oxidation (2), anti-bacterial (3), anti-cancer (4) and alleviation of immunotoxicity (5), relatively few studies have focused on FJR. Studies have revealed that FJR is rich in phenolic compounds, such as flavonoids, tannins, and coumarin derivatives, and exhibits many similar biological activities with walnuts (2, 6, 7). In the previous studies, FJR had proven to have a strong antioxidant ability and a certain anti-proliferation activity in cancer cells and displayed the ability to inhibit the growth of *Staphylococcus aureus* and *Escherichia coli*, and could alleviate diabetes in streptozotocin-induced diabetic mice (8–10). Due to its unique nutritional and medicinal value, FJR is often consumed as a green food and functional food in Southwest China (11).

It is well-recognized that the bioavailability of polyphenols depends on their bioaccessibility. According to previous research, polyphenols are not efficiently absorbed in the gastrointestinal tract, and most polyphenols end up in the colon, and then interact with the colonized gut microbiota (12). However, there are virtually no reports about the digestion stability, bioavailability and metabolic characteristics of FJR and its bioactive phytochemicals. In this sense, studying the release and catabolism of FJR subjected to *in vitro* digested and colonic fermented conditions is crucial. Although *in vivo* digestions best represent the digestion of food in real conditions, in recent years, the models of *in vitro* simulating digestion and colonic fermentation have been used as an alternative approach to explore the release and conversion of polyphenols from food, such as *Perilla frutescens* leaf and Lettuce (13, 14). Compared with the *in vivo* digestion method, the *in vitro* simulating digestion methods display many advantages such as low cost, rapid detection, and ease of use (15). In view of all the above facts, it is reasonable to investigate the bioactivities and bioaccessibility of FJR based on *in vitro* simulating digestion and colonic fermentation methods.

The release of bioactive compounds, antioxidant abilities, α-glucosidase inhibitory ability, α-amylase inhibitory ability and bioaccessibility of FJR under gastrointestinal digestion and colonic fermentation were studied in this work. Subsequently, the effects of FJR on the short chain fatty acids (SCFAs) produced during colonic fermentation and the microbiota composition in feces were also investigated. We expect that our results could provide a novel insight for the further development and utilization of FJR.

## Materials and methods

### Chemicals and reagents

The mature FJR was purchased from Dayao County in the Chinese province of Yunnan, in March 2020, which was identified as the flower of *Juglans regia* by Dr. Qi Jianhua, Forestry College, Southwest Forestry University. Folin-Ciocalteu reagent, gallic acid, rutin, 2,2-diphenyl-1-picrylhydrazyl radical (DPPH), 2,2'-azinobis (3-ethylbenzothiazoline-6-sulfonic acid) diammonium salt (ABTS), 2,4,6-tri (2-pyridyl)-1,3,5-triazine (TPTZ), p-Nitrophenol α-D-glycopyranoside (pNPG), vitamin C (Vc), potassium chloride (KCl), Dipotassium dipotassium hydrogen phosphate (K_2_HPO_4_), Magnesium magnesium chloride hexahydrate (MgCl_2_·6H_2_O), sodium chloride (NaCl), hydrochloric acid (HCl), ammonium carbonate (NH_4_)_2_CO_3_, 6-hydroxy-2,5,7,8-tetramethylchroman-2-carboxylic acid (Trolox), and 2,6-di-tert-butyl-4-methylphenol (BHT) were purchased from Sinopharm Chemical Reagent (Beijing, China). Acetonitrile (chromatographic grade) was purchased from Merck (Darmstadt, Germany). α-Glucosidase (100 U/mg, from Saccharomyces cerevisiae), α-amylase (50 U/mg, from hog pancreas), acarbose, P-nitrophenyl-α-D-glucopyranoside (pNPG) were purchased from Sigma-Aldrich (St. Louis, USA). The trypsin (1:250, from porcine gastric mucosa), pepsin (1:3,000, from porcine gastric mucosa) were purchased from Jiangsu Ruiyang Biotechnology Co. Ltd. (Jiangsu, China). HPLC standards (salicylic acid, epicatechin, 3,5-Dihydroxybenoic acid, etc.) were purchased from Yuanye Bio-Technology (Shanghai, China) and Merck (Darmstadt, German). SCFAs standards (acetic acid, propionic acid, isobutyric acid, butyric acid, isovaleric acid, valeric acid, and caproic acid) were purchased from Sigma-Aldrich (St. Louis, USA). All solvents used for the HPLC analysis were of HPLC grade. Other chemical reagents were of analytical grade.

### Sample preparation

To create powder samples, the FJR was cleaned with distilled water, dried at 45°C (thermostatic blast drying, DHG Series, Shanghai, China), pulverized (multifunction pulverizer, Redsun Electromechanical Co. Ltd., Yongkang, China), and sieved through a 60-mesh screen. For further analysis, samples were stored at −20°C.

#### *In vitro* gastrointestinal digestion

Based on the research of Xie et al. (16), a static *in vitro* digestive model was created to imitate oral, gastric, and intestine digestion. Firstly, powder samples were mixed with water at 1:30 (w/v), mixed thoroughly with simulated oral (0.65 mg α-amylase, pH 6.5). Then, the obtained mixture was incubated in a stirring water bath for 15 min at 37°C. After centrifuged centrifuging at 4,500 rpm, the mixture supernatants were obtained. In the gastric phase, the digested oral sample dispersed with deionized water (30 mL), then simulated gastric juice (30 mL, with 25 mg pepsin, pH = 2) was added. The mixture was heated to 37°C and agitated for 1 h. Followed by centrifugation and the supernatant was collected. For the final intestinal phase, 30 mL of distilled water and 30 mL of gastric sample were added to the 30 mL intestine digestion (4 mg trypsin, 30 mg bile salt, 54 mg NaCl, 6.5 mg KCl, pH = 7), followed by another 2 h of incubation at 37°C and 60 rpm. After the digestion was complete, it was centrifuged for 15 min and the supernatant was collected. The samples in different digestion stages (oral, gastric, and intestine digestion) were all collected and kept in storage at −20°C until further research.

#### *In vitro* colonic fermentation

The human fecal fermentation protocols were reviewed and approved by the Academic Committee of Southwest Forestry University (ethical approval number SWFU-2021015). Firstly, fecal samples were gained from three healthy Chinese volunteers (one male, two females, mean age = 23.7). All of the following requirements had to be met by them: (1) Had not taken any antibiotics for at least 3 months; (2) Good eating habits; (3) No digestive system illnesses. Fecal samples were mixed in a ratio of 1:1:1 and transported right away to the anaerobic chamber. Sterile phosphate buffered saline (w/v, 0.1 M, pH 7.2) was added to the fecal samples. A sterilized glass rod was used to stir each mixture for 5 min, and filtered through 2 layers of cheesecloth.

Following that, 0.005 g FeSO_4_·7H_2_O, 0.08 g CaCl_2_, 0.4 g bile acid, 0.5 g K_2_HPO_4_, 0.69 g MgSO_4_·H_2_O, 0.8 g L-cysteine, 1 g guar gum, 1.5 g NaHCO_3_, 2 g arabinogalactan, 2 g pectin, 3 g casein, 4 g mucoprotein, 4.5 g KCl, 4.5 g NaCl, 4 mL resazurin (0.025 %, w/v), and 1 mL Tween 80 were dissolved in 1 L deionized water to prepare a basic medium (17). Fecal slurry (10%, w/w) was mixed with fermentation medium (The post intestinal digestion samples) in a 1:1 ratio, and incubated at 37°C for 48 h under anaerobic conditions. Colonic fermentation was carried out in the absence without adding anything in the control group. For further analysis, samples were taken at 0, 6, 12, 24, and 48 h. The pH measurement of each time period was carried out with a pH meter (Hanna Instrument^®^, Ann Arbor, Michigan, USA). The samples were cooled and kept at 4°C for 20 min. The supernatants were then gathered and stored at −80°C in preparation for analysis.

### Determination of total phenolic content and total flavonoid content

The total phenol content (TPC) of FJR during digestion and fermentation was quantified according to Folin–Ciocalteu analysis by measuring the absorbance of the solution at 765 nm with a microplate reader (18). Briefly, 50 μL of properly diluted samples or standard at different concentrations were mixed with 125 μL of Folin–Ciocalteau reagent (10%) in a 96-well microplate. Next, 100 μL of Na_2_CO_3_ (7.5%, w/v) was added to the mixtures. The reaction was then held at room temperature for 30 min in the dark. The data were expressed as μg of gallic acid equivalents (GAE)/mg extract (DE).

Total flavonoid content (TFC) was measured by the aluminum nitrate colorimetric method (19). Forty μL of properly diluted sample was mixed with 20 μL of NaNO_2_ (3%, w/v). After 6 min of reaction, 20 μL of Al (NO_3_)_3_ (6%, w/v) was added, and the mixture was incubated for another 6 min. After that, 140 μL of NaOH (4%, w/v) and 60 μL of 70% methanol were added. After the combination solution had stood for 15 min, and the absorbance was measured at 510 nm. Rutin was used as the standard reference, and the final result was expressed as μg of rutin equivalents (RE)/mg extract (DE).

### Antioxidant assays

The antioxidant abilities were evaluated by three different methods (ABTS^+^, DPPH·, and FRAP). The absorbance readings were measured using a Universal microplate reader (pectramax PLUS 384, Molecular Device, San Jose, CA, USA).

ABTS Radical Scavenging Ability: ABTS radical scavenging ability of FJR and its digestive products were determined by previously reported method (20). In brief, after incubating ABTS^+^·working solution (200 μL) with sample solutions (50 μL) for 5 min in a 96-well plate, the absorbance was measured at 734 nm. Butylated hydroxytoluene (BHT) and ascorbic acid were used as the reference standard. Results were expressed microgram Trolox equivalents per milligram of sample dry extract (μg TE/mg DE).

DPPH Radical Scavenging Ability: The free radical scavenging ability of FJR samples was evaluated according to our previously published paper by Wang (13). The suitable concentration of sample (100 μL) was mixed with 100 μL DPPH working solution in a 96-well microplate, then the mixtures were kept at room temperature in the dark for 30 min. BHT and ascorbic acid were used as the reference standard. Results were expressed microgram Trolox equivalents per milligram of sample dry extract (μg TE/mg DE).

The ferric reducing antioxidant power (FRAP) assay: FRAP was quantified by the reported method (21). Properly diluted samples (50 μL) were mixed with 250 μL of freshly prepared FRAP working solution (10 mmol/L TPTZ, 20 mM FeCl_2_, and 300 mM acetate buffer were prepared in the ratio of 1:1:10) in a 96-well microplate. After incubation in darkness for 10 min at 37°C, the absorbance was measured at 593 nm. Vc and BHT were used as positive controls. The FeSO_4_ was used as the standard and the results were expressed as μg of FeSO_4_ equivalents/mg extract (DE).

### α-glucosidase inhibitory ability

The α-glucosidase inhibitory ability was measured based on the previously reported method (19), with slight modifications. Briefly, 50 μL of samples was mixed with 50 μL of the α-glucosidase solution (0.1 U/mL) and incubated in a 96-well plate at 37°C for 10 min. After probation, 50μL of pNPG was added. The reaction was carried out at 37°C for 15 min, then the reaction was stopped by adding Na_2_CO_3_ (100 μL 0.2 mol/L). The absorbance was recorded at 405 nm. Acarbose was positive control, and the results were expressed as micrograms acarbose equivalent per milligram of extract (μg GAE/mg).

### α-amylase inhibitory ability

The α-amylase inhibitory ability was evaluated by the dinitrosalicylic acid (DNS) method. Briefly, 20 uL of samples was mixed with 20 uL of the α-amylase solution and incubated in a 96-well plate at 25°C for 10 min. Subsequently, 100 μL soluble starch was added and the reaction mixture was incubated at 25°C for 10 min, followed by the immediately adding of 20 μL 3,5-dinitrosalicylic acid (DNS) color reagent to stop the reaction. Finally, the micro-well plates were placed in a boiling water bath for 10 min and then cooled to room temperature. After cooling at room temperature, 100 μL distilled water was added. The absorbance was recorded at 540 nm. Acarbose was positive control, and the results were expressed as micrograms acarbose equivalent per milligram of extract (μg GAE/mg).

### HPLC-DAD analyses

The contents of 16 phenolic compounds (Supplementary Table 1) in FJR were quantified by HPLC-DAD analysis (Agilent Technologies, CA, USA). Samples were filtered through 0.22 μm nylon syringe filters for HPLC analysis. The C18 reversed phase analytical column (250 mm × 4.6 mm, 5 μm, Greenherbs Science and Technology, Beijing, China) was maintained at 25°C, samples were scanned from 200 to 400 nm, with 0.1% formic acid (A) and acetonitrile (B) as mobile phase with a flow rate of 0.8 mL/min. The gradient elution conditions were: 0 min, 5% B; 5 min, 5% B; 7 min, 10% B; 52 min, 30% B; 65 min, 100% B; 70 min, 100% B; 80 min, 5% B.

### Short chain fatty acids assays

Short chain fatty acids (SCFAs) were quantified using Gas chromatography-mass spectrometry (GC-MS) analysis. Thirty mg of the post colonic fermentation samples and 900 μL of phosphoric acid solution (0.5%) were added to the placed in a 2 mL glass centrifuge tube and then centrifuged at a high speed of 14,000 rpm for 10 min, for further GC-MS analysis. Later, 600 uL of the organic phase were taken and 4-methyl valeric acid as the internal standard (500 μM). The injection volume was 1 L, and the split ratio was 10:1. The fatty acids were calculated based on a standard curve.

### 16S rRNA gene sequencing analysis

Extraction of total bacterial DNA from frozen stool samples was done using the Fecal DNA kit according to the instruction manual, and sent to Shanghai Applied Protein Technology (Shanghai, China) for the microbial analysis. The V3–V4 region of the 16S rRNA gene was amplified with the former 341F (CCTAYGGGRBGCASCAG) and the reverse 806R (GGACTACNNGGGTATCTAAT) and then sequenced using the Illumina Miseq platform (IIIumina, San Diego, USA). Quantitative Insights Into Microbial Ecology (QIIME, v1.8.0)33 was used to process the raw read sequences.

### Statistical analyses

All experiments were done in triplicate and expressed as mean ± standard deviation. Graphing and statistical analysis were performed by Origin 2018 software and SPSS Version 22.0. software, respectively. *P* < 0.05 was considered significant. PCA and correlations were generated with R software (version 4.0.6).

## Results

### Total phenolic and flavonoid content

**Figure 1:**
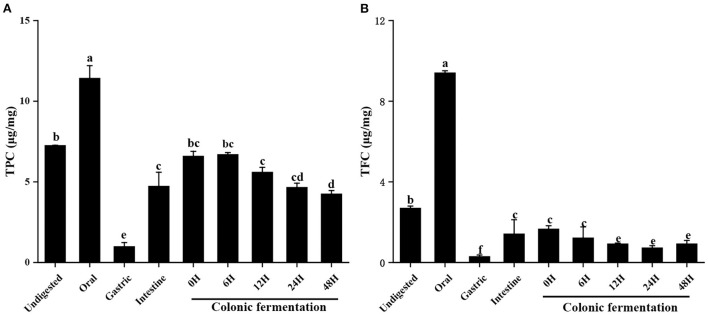
Contents of total phenols and total flavonoids of FJR in simulated digestion colonic fermentation stage in vitro. **(A)** Total phenolic content. **(B)** Total flavonoid content. Different letters (a-e) mean significant difference (*p* < 0.05).

The TPC and TFC throughout the digestion and colonic fermentation were depicted in Figure 1. As can be observed, the different phase *in vitro* digestion affected differently TPC and TFC in FJR. TPC and TFC in the digested after the oral step were 11.43 ± 0.77 μg/mg and 9.41 ± 0.10 μg/mg, respectively. Compared to the initial content of the undigested FJR, they did significantly higher (*p* < 0.05). While in gastric, the TPC and TFC of FJR were only 0.99 ± 0.24 μg/mg and 0.30 ± 0.07 μg/mg, which were a statistically significant decrease of 86 and 89%, respectively, in comparison with the undigested control *(p* < 0.05). Experimental data revealed that TPC and TFC released by the *in vitro* colonic fermentation process from FJR were higher than the gastric digestion process. According to Figure 1, TPC and TFC increased continuously as fermentation time increased from 0 to 6 h, supporting past research that alkaline hydrolysis to be more effective than acid hydrolysis at releasing phenolic compounds (22). However, compared to the 6 h fermentation, the TPC and TFC were much lower during the 48 h colonic fermentation. A possible reason for the decrease was that the substances in FJR were utilized, degraded, and transformed by microorganisms during the fermentation (23).

Overall, the results demonstrated that the contents of total flavonoids and total polyphenols in oral and intestine were relatively higher than gastric. Which indicated most of the loss of TPC and TFC was attributable to the alteration in the chemical environment during digestion. Under the acidic environment of the stomach, some phenolics and flavonoids were degraded (24). In contrast, the action of intestinal microbes increased the released amount of phenolics and flavonoids in the intestine (25). On the other hand, the oral almost had higher TPC and TFC values than each phase (*p* < 0.05). The reason might be that the solubilisation of phenolic compounds and flavonoids may have increased as a result of stirring or enzyme ability (α-amylase) during oral simulation. Both of which may have resulted in the release of phenolic contents and flavonoids (26).

### Antioxidant abilities

**Figure 2:**
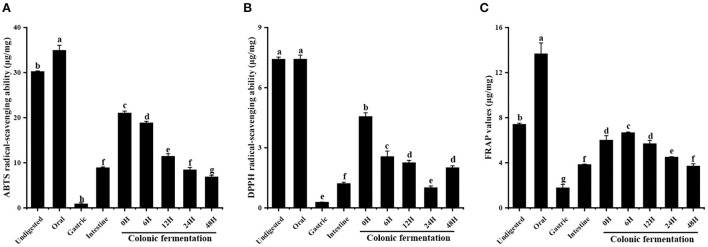
Antioxidant abilities of FJR in simulated digestion colonic fermentation stage *in vitro*. **(A)** ABTS radical scavenging ability. **(B)** DPPH radical scavenging ability. **(C)** FRAP. Different letters (a-h) mean significant difference (*p* < 0.05).

The antioxidant ability of FJR active compounds may be greatly impacted by its instability during digestion. Thus, in order to assess the antioxidant ability of undigested and digested samples, three methods (ABTS^+^, DPPH·, and FRAP) were used in this work. Of which DPPH and ABTS radical scavenging assays utilize hydrogen atom transfer and single electron transfer reaction mechanisms, while the FRAP assay takes up the single electron transfer method. As the digestive process progressed, the scavenging of ABTS (Figure 2A) was the highest in oral (34.97 ± 1.09 μg/mg) and lowest in gastric (0.91 ± 0.21 μg/mg). After intestinal digestion, the ABTS scavenging ability of FJR was 8.91 ± 0.17 μg/mg, which was much lower than undigested, but remained significantly higher than gastric. The FJR after 0–48 h of colonic fermentation showed continuous decreased ABTS radical scavenging ability of 21.05 ± 0.41 to 6.87 ± 0.36 μg/mg.

Similar behavior was observed when DPPH was evaluated (Figure 2B). The oral digestion product of the FJR displayed the highest DPPH radical-scavenging ability (7.43 ± 0.10 μg/mg), which was higher than that of undigested fractions, but there were no statistically significant differences among them (*p* > 0.05). Followed by intestine digestion (1.22 ± 0.06 μg/mg) and gastric digestion (0.28 ± 0.01 μg/mg). The DPPH radical scavenging ability declined from 4.56 ± 0.18 to 1.01 ± 0.08 μg/mg during 0 h to 24 h of colonic fermentation and then increased to 2.01 ± 0.09 μg/mg after 48 h.

After undergoing the oral phase compared to those undigested, a significantly larger FRAP value was observed in the FJR samples (Figure 2C), the values increased from 7.42 ± 0.10 μg/mg to 13.68 ± 0.96 μg/mg. Similar to the ABTS and DPPH, the lowest FRAP value was observed in the gastric phase. In the colonic fermentation phase, the FRAP increased first and then decreased, with the highest value of 6.68 ± 0.05 μg/mg at fermented 6 h, and the lowest value of 3.72 ± 0.20 μg/mg at fermented 48 h, which were significantly different from each other (*p* < 0.05).

**Figure 3:**
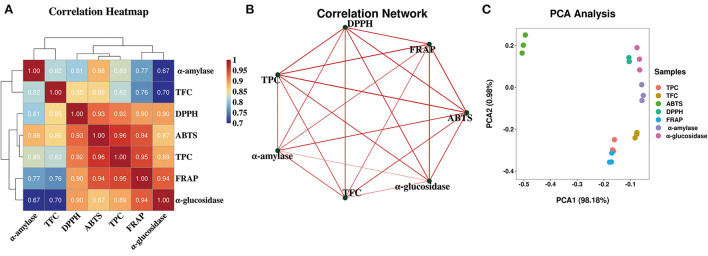
Heatmap graph of correlation analysis and principal component analysis (PCA) of diferent samples and indicators. **(A)** Correlation heatmap. **(B)** Correlation network. **(C)** PCA analysis. *P*-value (ranging from 0 to 1) and corresponding color (red to blue) represented the magnitude of the Pearson correlation.

Over all, the antioxidant abilities showed similar trends with TPC and TFC. While these three antioxidant abilities were significantly decreased after gastrointestinal digestion, they still maintained certain antioxidant abilities after colonic fermentation when compared with undigested samples. To elucidate the correlation between TPC, TFC, antioxidant abilities, and the other variables more clearly, the correlation analysis and principal component analysis (PCA) were further performed and were displayed in Figure 3 in the form of the correlation heatmap and PCA graph, respectively. The correlation analysis showed a good correlation between TPC of FJR and the changes in ABTS radical scavenging ability (*R*^2^ = 0.96), DPPH scavenging ability (*R*^2^ = 0.90), and the FRAP (*R*^2^ = 0.95) during each stage of digestion. This revealed that antioxidant abilities were relevant to the release content, biological activity, stability, and bioavailability of FJR after digestion. The PCA results exhibited that each group can be well-differentiated and the relative distance between TPC and antioxidant abilities indicated the higher correlations between.

### The α-glucosidase and α-amylase inhibitory abilities

α-Glucosidase and α-amylase, which are the two key enzymes considered pharmacological targets in anti-diabetic therapy. Therefore, one of the effective strategies to control postprandial hyperglycemia is delaying glucose absorption using α-glucosidase and α-amylase inhibitors (27). The study measured the α-glucosidase and α-amylase inhibitory abilities of FJR during the whole digestion process.

**Figure 4:**
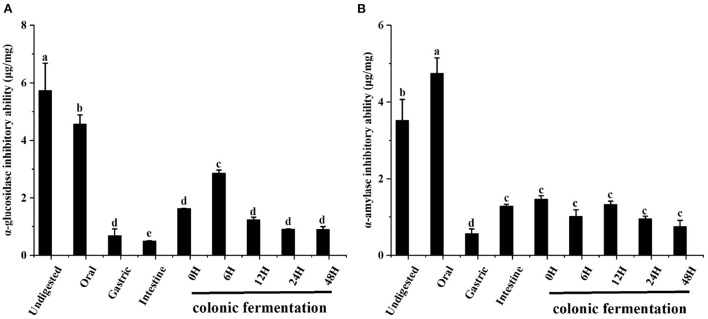
The α-glucosidase and α-amylase inhibitory abilities of FJR in simulated digestion colonic fermentation stage *in vitro*. **(A)** α-glucosidase inhibitory ability. **(B)** α-amylase inhibitory ability. Different letters (a-e) mean significant difference (*p* < 0.05).

The α-glucosidase inhibitory ability of FJR was shown in Figure 4A. When compared to the undigested stages (5.73 ± 0.96 μg/mg), the inhibitory ability of FJR continuous reduction to 0.49 ± 0.02 μg/mg. In contrast, the inhibitory ability of colonic fermentation phase presents an overall trend of increasing first and then decreasing, which achieved the highest at 6 h (2.86 ± 0.12 μg/mg). The above results showed that the oral and gastrointestinal digestion process negatively influence the ability of α-glucosidase inhibitory ability. These results were consistent with previous reports that gastrointestinal digestion decreased α-glucosidase inhibitory ability of FJR (28). It was primarily speculated that the destruction of the main compounds related to the abilities during digestion may be responsible for the reduction of inhibitory. Whereas, during colonic fermentation, the substances in FJR were utilized, degraded, and transformed into compounds with inhibitory ability by microorganisms. So that the ability to inhibit α-glucosidase inhibitory ability increases during short-term colonic fermentation (0–6 h).

The changes in the α-amylase inhibitory ability during the simulated gastrointestinal digestion and colonic fermentation processes are presented in Figure 4B. In the oral digestion, α-amylase showed different results from α-glucosidase, the inhibitory ability has increased significantly, which was 4.74 ± 0.41 μg/mg. After gastric digestion, α-amylase inhibitory was the weakest (0.56 ± 0.13 μg/mg). At the end of the intestinal phase, the changes in the α-amylase inhibitory ability were smaller and the trend was not statistically significant (*p* > 0.05).

From these results, it can be concluded that the α-glucosidase and α-amylase inhibitory abilities tend to follow a different changes form along the digestion and colonic fermentation processes of FJR. For α-amylase inhibitory ability, the variation trends coincide with that of TPC, TFC and antioxidant abilities. Many substances with α-amylase inhibitory ability will be released during oral digestion. Conversely, colonic fermentation had little impact on α-amylase inhibitory ability. The correlation analysis and PCA analysis further validated that α-glucosidase and α-amylase inhibitory abilities were significant correlated with TPC/TFC (*p* < 0.01), which demonstrated that TPC/TFC were the material basis for the inhibition of α-glucosidase and α-amylase. In conclusion, FJR still maintained some α-glucosidase and α-amylase inhibitory abilities after *in vitro* gastrointestinal digestion and fecal fermentation, indicating certain hypoglycemic potential.

### HPLC-DAD analysis

**Figure 5:**
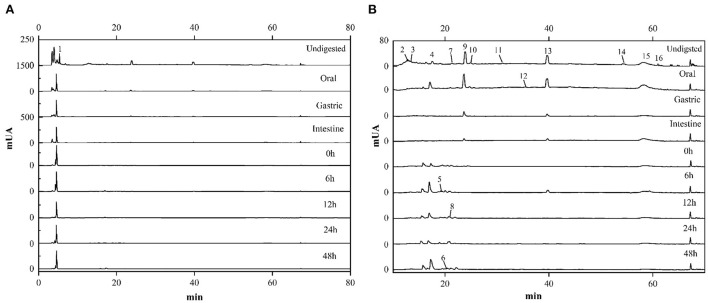
HPLC chromatograms of FJR during in vitro simulated digestion and colonic fermentation. **(A)** Chromatogram at the larger ordinate (0-1500). **(B)** Chromatogram at the smaller ordinate (0-80).

In order to elucidate the change of the various compounds in FJR during the simulated gastrointestinal digestion and colonic fermentation processes, HPLC analysis was performed to present their chromatographic characterizations. According to the previous study (2), corresponding standards were selected, and 16 compounds were detected. The quantification of 13 phenolic acids and 3 flavonoids at different digestion phases and fermentation stages was shown in Table 1. These compounds were labeled in (Figures 5A,B) with Arabic numerals according to their elution order.

**Table 1:** HPLC analysis results of FJR during *in vitro* simulated digestion and colonic fermentation.

**Number**	**Compounds**	**Species**	**Undigested (ug/mg)**	**Oral (ug/mg)**	**Gastric (ug/mg)**	**Intestine (ug/mg)**	**0 h (ug/mg)**	**6 h (ug/mg)**	**12 h (ug/mg)**	**24 h (ug/mg)**	**48 h (ug/mg)**
1	Gallic acid	Phenolic acids	0.009 ± 0.003	–	–	–	–	–	–	–	–
2	Protocatechuic acid	Phenolic acids	0.140 ± 0.008^c^	0.675 ± 0.013^a^	0.082 ± 0.000^c^	–	–	0.317 ± 0.114^b^	–	–	–
3	3,5-Dihydroxybenzoic acid	Phenolic acids	0.326 ± 0.032	–	–	–	–	–	–	–	–
4	p-Hydroxybenzoic acid	Phenolic acids	0.016 ± 0.002^e^	0.015 ± 0.003^e^	–	–	0.063 ± 0.005^c^	0.210 ± 0.019^b^	0.032 ± 0.009^a^	0.042 ± 0.010^cd^	0.026 ± 0.005^de^
5	2,5-Dihydroxybenzoic acid	Phenolic acids	–	–	–	–	0.068 ± 0.003^c^	0.418 ± 0.015^a^	0.273 ± 0.023^b^	–	–
6	p-Coumaric acid	Phenolic acids	–	–	–	–	–	–	–	–	0.050 ± 0.001
7	Vanillic acid	Phenolic acids	0.260 ± 0.001^c^	0.266 ± 0.001^c^	–	–	0.061 ± 0.005^d^	0.471 ± 0.040^b^	0.521 ± 0.007^a^	0.469 ± 0.007^b^	0.046 ± 0.026^d^
8	Caffeic acid	Phenolic acids	–	–	–	–	–	–	0.031 ± 0.014^b^	–	0.140 ± 0.001^a^
9	Epicatechin	Phenolic acids	0.609 ± 0.013^a^	0.124 ± 0.002^d^	0.429 ± 0.009^b^	0.183 ± 0.010^c^	0.055 ± 0.009^e^	–	–	–	–
10	Dihydromyricetin	Flavonoids	0.078 ± 0.001^a^	0.055 ± 0.005^b^	–	–	–	–	–	–	–
10	Ferulic acid	Phenolic acids	0.042 ± 0.001^b^	0.057 ± 0.005^a^	–	–	–	–	–	–	0.030 ± 0.002^c^
12	Ellagic acid	Phenolic acids	–	0.383 ± 0.003	–	–	–	–	–	–	–
13	Salicylic acid	Phenolic acids	1.040 ±0.054^b^	1.241 ± 0.071^a^	0.409 ± 0.098^d^	0.184 ± 0.003^4^	–	0.703 ± 0.015^c^	–	–	–
14	Cinnamic acid	Phenolic acids	0.037 ± 0.001	–	–	–	–	–	–	–	–
15	Hesperetin	Flavonoids	0.039 ± 0.001^b^	0.037 ± 0.001^b^	–	–	–	0.284 ± 0.007^a^	–	–	
16	Baicalein	Flavonoids	0.034 ± 0.001^b^	0.039 ± 0.004^b^	–	–	–	0.170 ± 0.009^a^	–	–	–

a−*e* Mean values in the same row indicated the significantly different (*p* < 0.05).

Overall, the contents of each compound in the FJR varied along with digestion processes. In terms of content, phenolic acids were the main compounds followed by flavonoids. Of those, salicylic acid was the main compound of FJR, it was almost detected in each stage. Undigested and the oral digestion stage are identified the most compounds, mainly salicylic acid, epicatechin, 3,5-dihydroxybenoic acid, vanillic acid, and protocatechuic acid. In contrast, the identified compounds from the gastric and intestine digestion stage were less abundant, only protocatechuic acid, epicatechin and salicylic acid were found. However, compared with oral digestion, the released protocatechuic acid and salicylic acid from gastric digestion were decreased by 87.86, and 85.17%, respectively, while the released epicatechin was increased. The results of the experiments shown, the contents of compounds retained after digestion and reached the colon were significantly higher than those absorbed by the small intestine. Similar results were found in experiments on passion fruit peel (29). Besides phenolic acids, flavonoids also suffered an intense degradation after digestion. Dihydromyricetin (0.078 ± 0.001 μg/mg) was one of the main flavonoids detected in the FJR. However, in flavonoids, only hesperetin (0.037 ± 0.001 μg/mg), and baicalein (0.034 ± 0.001 μg/mg) were present after oral digestion, which both of their contents also decreased along the digestion. Surprisingly, both of them were detected in the colonic fermentation of 6 h, which were 0.284 ± 0.007 and 0.170 ± 0.009 μg/mg, respectively.

Moreover, ellagic acid was not detected in undigested samples, but it was released during the oral phase (0.383 ± 0.003 ug/mg). In fact, except for ellagic acid, protocatechuic acid, vanillic acid and salicylic acid were also released increased during the oral phase, which were increased by 79.26, 2.26, and 19.20%, respectively. We also noticed that p-coumaric acid and caffeic acid were detected only during colonic fermentation but not in gastrointestinal digestion. These data indicate that these compounds in the fermentation supernatants likely came from microbial transformation or other compounds.

**Figure 6:**
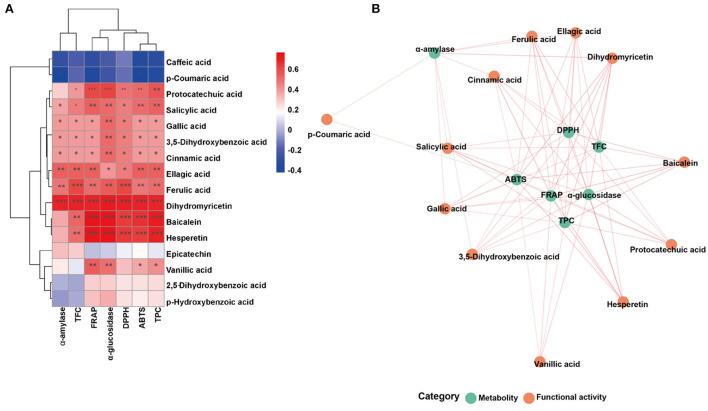
Associated network diagram and correlation heat map of spearman's analysis shows the correlation between metabolites and functional activities. **(A)** Associated network of FJR. **(B)** Correlation heat map of spearman's analysis of FJR. Red lines represent positive correlations while green lines represent negative correlations between metabolites (green circle) and functional activities (red circle). *p < 0.05, **p ≤ 0.01, and ***p ≤ 0.001.

Furthermore, the correlations were assessed using Spearman's method and presented as network diagrams and heat maps to further study the relationship between metabolites and sixteen functional activities in FJR (Figure 6). Among Spearman's algorithms, the TPC, ABTS, DPPH, FRAP, and α-glucosidase showed a strong correlation with hesperetin and baicalein. Additionally, all functional activity showed a strong correlation between the dihydromyricetin, while two metabolites (p-coumaric acid, caffeic acid) showed significantly negatively correlated with all activity indicators. It can be concluded that functional activities of FJR may be regulated by a variety of active substances. Among them, hesperetin, baicalein, and dihydromyricetin were the primary active substances. Figure 6B further highlights the intricate connections between these functional activities and important metabolites.

### Short chain fatty acids analysis

SCFAs are the major class of essential microbial metabolic products in the large intestine and colon, which can be rapidly absorbed by enterocytes or released from the gut. It is an important indicator of intestinal homeostasis and has a strong link between human health (30, 31). In recent years, the notion that SCFAs are metabolic targets was broadly accepted (32, 33). Therefore, the SCFAs production during fermentation (F0 h, F6 h, F24 h) was determined *via* the GC method to evaluate the potential effect of FJR on the gut microenvironment, and without adding FJR was used as a control (FC6 h, FC24 h). Based on this, seven SCFAs were effectively separated in the GC column, including acetic acid, propionic acid, iso-butyric acid, butyrate, isovaleric acid, pentanoic acid, and caproic acid (Table 2). The result shows that during the experimental period, the total SCFAs in the colonic fermentation groups were mostly greater than the control group. Among them, at 24 h after fermentation, total SCFAs content (F24 h) was the highest at 21,208.88 ± 3,150.18 μg/g, followed by 6 h (F6 h), whereas unfermented (F0 h) was the lowest (4,634.32 ± 745.13 μg/g). The total SCFAs content of the control group was the highest at 24 h (FC24 h), which was 20,268.89 ± 268.35 μg/g. Furthermore, the FC6 h sample from the control group had the lowest total SCFA content, but it was still higher than the sample without fermentation (F0 h) (*p* < 0.05). This reason might be that gut microbes generate SCFAs by fermentation of dietary fibers in FJR, which is reported by several similar studies previously (34–36).

**Table 2:** Content of short-chain fatty acids in colonic fermentation of FJR.

**Sample**	**Time**	**Acetic acid (μg/g)**	**Propionic acid (μg/g)**	**Iso-butyric acid (μg/g)**	**Butyrate (μg/g)**	**Isovaleric acid (μg/g)**	**Pentanoic acid (μg/g)**	**Caproic acid (μg/g)**	**Total content (μg/g)**
FJR	0 h	2,989.13 ± 416.17^b^	694.65 ± 121.67^a^	65.54 ± 23.92^a^	692.46 ± 152.62^a^	76.3 ± 12.86^a^	110.66 ± 19.72^a^	5.56 ± 1.26^a^	4,634.32 ± 745.13^c^
	6 h	7,969.49 ± 1117.60^b^	581.86 ± 107.60^a^	56.71 ± 16.05^a^	570.79 ± 118.29^ab^	62.66 ± 16.96^a^	92.31 ± 22.09^ab^	4.27 ± 0.81^ab^	9,338.08 ± 1,349.16^b^
	24 h	20,119.59 ± 2873.39^a^	510.51 ± 106.65^a^	32.14 ± 9.43^b^	448.69 ± 133.30^b^	36.10 ± 13.23^b^	59.19 ± 19.44^b^	2.66 ± 0.82^b^	21,208.88 ± 3,150.18^a^
Control	FCC6 h	7,255.89 ± 308.17^ab^	648.37 ± 52.73^a^	66.23 ± 9.07^a^	676.73 ± 63.11^a^	74.10 ± 10.19^a^	112.08 ± 14.06^a^	4.51 ± 0.62^a^	8,768.95 ± 382.02^b^
	FC24 h	19,037.54 ± 242.08^a^	575.02 ± 10.22^a^	42.61 ± 1.20^ab^	491.94 ± 13.34^ab^	52.27 ± 1.88^ab^	66.84 ± 2.20^b^	2.66 ± 0.11^b^	20,268.89 ± 268.35^a^

a−*c* Mean values in the same row indicated the significantly different (*p* < 0.05).

Additionally, the concentrations of acetic acid, propionic acid, and butyrate still remained at a higher level after 24 h colonic fermentation. In particular, accumulation of acetic acid in the fermentation group (F24 h) was much higher than that in the control group (F0 h) (*p* < 0.05), with a peak concentration of 20,119.59 ± 2,873.39 μg/g. The study indicates that acetic acid can improve glucose tolerance and restore glucose-induced insulin secretion HF mice (37). Propionic acid would affect the liver and cholesterol metabolism, could regulate FFA3 pathway to protect the liver by decreasing hepatic glucose production (38). During fermentation, the overall trends of variations in the propionic acid, iso-butyric acid, butyrate, isovaleric acid, pentanoic acid and caproic acid contents during the fermentation process were consistent, and the highest contents were observed at 0 h (F0 h) and the lowest contents were observed at 24 h (F24 h). However, in general, the content of total SCFAs increased significantly with the extension of fermentation time, and the experiment's upward trend is more acute than the control's.

### pH values

**Figure 7:**
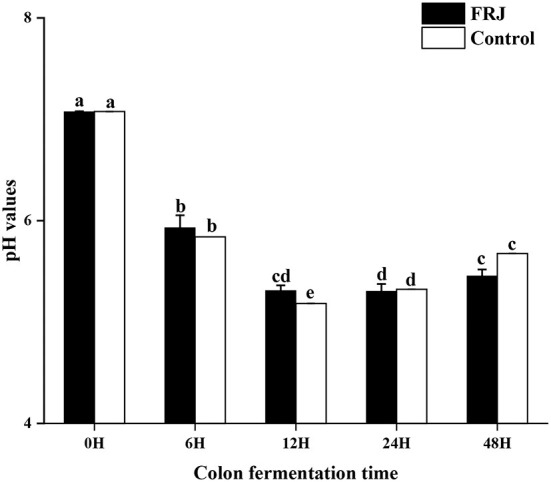
pH value of the colonic fermentation stage. Different letters (a-e) mean significant difference (*p* < 0.05).

The change of pH is one of the key indicators indices in the simulated human fecal fermentation model, which can reflect the fermentation process and degree and assess the performance of FJR. During colonic fermentation, its pH changes as depicted in Figure 7. After 12 h of incubation, a significant decrease of pH (the average value from 7.08 to 5.25) was observed for the fermentation in both groups (*p* < 0.05). However, during 6–24 h of fermentation, the pH of each group slightly increased (*p* < 0.05), but it still in a weak acid background. Interestingly, the pH value of the FJR group was mostly lower than that of the control group at the same fermentation time, which might be correlated with the higher level of SCFAs generated in the FJR group (Table 2) (39). Overall, the results indicated that FJR could decrease the pH of the contents of colonic fermentation.

### Gut microbiota analysis

**Figure 8:**
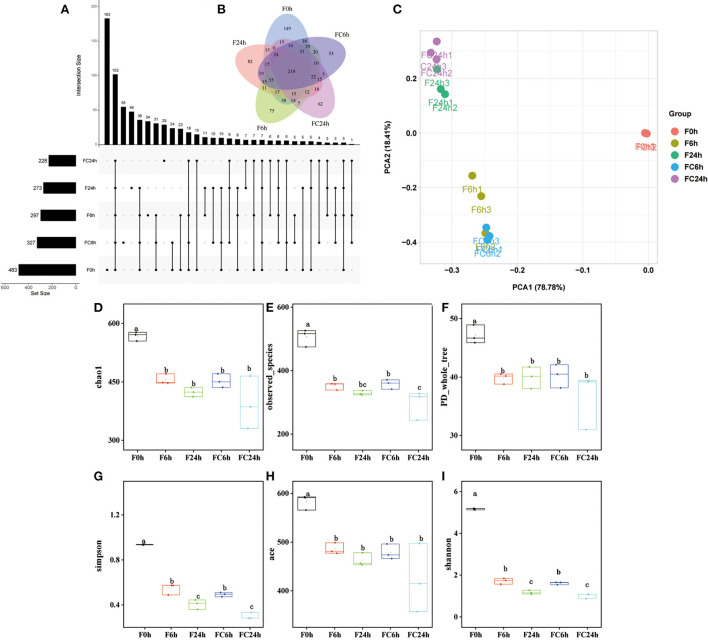
Alpha diversity indices boxplot among groups. **(A)** Upset plot; **(B)** Venn diagram; **(C)** PCA analysis; **(D)** Chao index; **(E)** Observed species; **(F)** PD whole tree; **(G)** Simpson index; **(H)** ACE index; **(I)** Shannon index; F0 h = Fermentation for 0 h with FJR; F6 h = Fermentation for 6 h with FJR; F24 h = Fermentation for 24 h with FJR; FC6 h = Fermentation for 6 h without FJR; FC24 h = Fermentation for 24 h without FJR. Different letters (a–c) mean significant difference (*p* < 0.05).

The microbiota in the human gut has a significant impact on health (40). The nutrient acquisition and energy regulation are also related to the composition of gut microbiota (41, 42). Understanding the connections between FJR, gut microbiota and bacterial metabolites can be beneficial for health improvement and disease prevention through modulating the gut microbiota. According to the pH values, it maintained a decreased trend after colon fermentation for 6 h and then remained stable at 24 h. Therefore, we further used high-throughput sequencing of the bacterial 16 S rRNA gene to determine the microbiota composition of feces at various fermentation sites (0, 6, 24 h), and without adding FJR was used as a control, in order to better examine the changes in fecal microbiota community structure from FJR. In general, sequences with at least 97% similarity were clustered into community richness (OTUs), which can represent the community richness. For OTUs analysis, there were 483, 327, 297, 273, and 228 OTUs for the F0 h, FC6 h, F6 h, F24 h, and FC24 h, respectively (Figure 8A). There was a steady decrease in the OTU richness of the entire community as the fermentation time increased, of which the number of OTUs for the F24 h was more than FC24 h, of which 218 OTUs were shared by these groups (Figure 8B). Next, we sought to quantify differences in gut microbiota compositions with PCA. According to Figure 8C, there was a highly significant difference between the 0, 6, and 24 h groups. However, the F6 h and FC6 h groups, F24 h and FC24 h were rather close to one another, which indicated more similar microbial community structures between samples. According to the α-diversity analyses (Figures 8D–I), FJR exhibited higher α-diversity than the control group. The results above both indicated that FJR played a positive role in maintaining community richness and diversity. FJR has the ability to change the microbial community structure, and its fermentation time will also have a great impact on the microbial composition.

**Figure 9:**
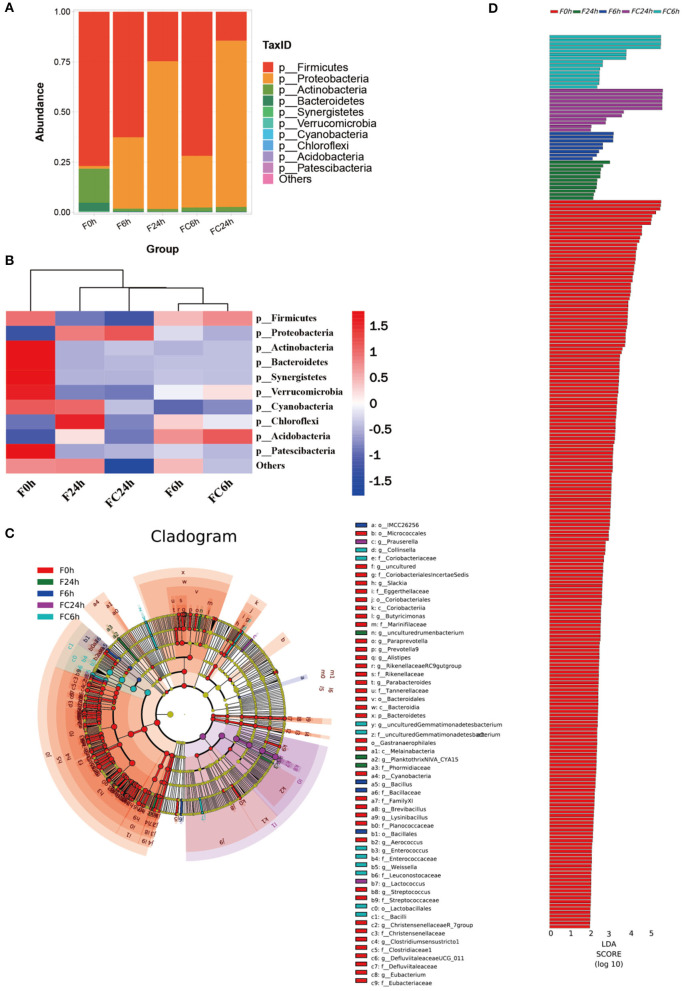
Effects of FJR on the structure of the gut microbiota composition *in vitro* fecal fermented at different times. **(A)** Microbial composition at phylum level during *in vitro* fecal fermentation; **(B)** The heatmap of the gut microbiota in all groups at the phylum level; Histogram **(C)** and cladogram **(D)** from LEfSe analysis. F0 h = Fermentation for 0 h with FJR; F6 h = Fermentation for 6 h with FJR; F24 h = Fermentation for 24 h with FJR; FC6 h = Fermentation for 6 h without FJR; FC24 h = Fermentation for 24 h without FJR.

The results of the evaluation of the microbial community structure at the phylum level are depicted in Figure 9A. Firmicutes, Actinobacteriota, and Bacteroidetes were the dominant bacteria in samples before fermentation (0 h). After fermentation (6 and 24 h), the gut microbiota composition of all the groups changed significantly. Among them, the levels of Firmicutes and Proteobacteria made up the majority of the bacteria in all groups. This conclusion was consistent with other reports that more than 90% of the bacteria in the colon belonged to the Firmicutes and Proteobacteria communities (43). The relative abundance of Proteobacteria, Actinomycetes dropped, and the relative abundance of Firmicutes did not change significantly in the control group at 6 and 24 h. Compared with the control group, the levels of the experimental group, Firmicutes, Bacteroidea and Actinobacteria Bacteroidota in the 6 and 24 h have reduced to varying degrees, while the relative abundances of Proteobacteria was significantly increased. It was reported that the growth inhibition of Firmicutes might be related to that of polyphenols and their metabolites (12), which was also in agreement with the results in Figure 1. Additionally, a series of beneficial bacteria such as *Bacteroidetes* and *Firmicutes* have a positive contribution to the production of SCFAs. This also explains the reason for the continued increase of SCFAs (44).

Heatmap of species composition at the phylum level as shown in Figure 9B. The proteobacteria increased during colonic fermentation (*P* < 0.05), but the F24 h was rise less than the FC24 h, which might be due to the inhibitory effect of caffeic acid (Table 1) from FJR on pathogens. In addition to proteobacteria, the chloroflexi in the F24 h was also significantly higher than that in the F0 h (Figure 9B). Overall, during gut fermentation, the microbiota in the gut may interact with polyphenols, bio-transform them, and produce a number of metabolites. Polyphenols themselves may also affect the balance of the microflora. The bioactivity of polyphenols might play an important role in the maintenance of colonic health. Taxonomic cladogram produced from LEfSe analysis shows the significant differences in microbiota communities among five groups, which indicates that each group with significantly differential abundance (Figure 9D). Furthermore, OTUs in 0 h was higher than other groups based on the LDA scores (Figure 9C).

**Figure 10:**
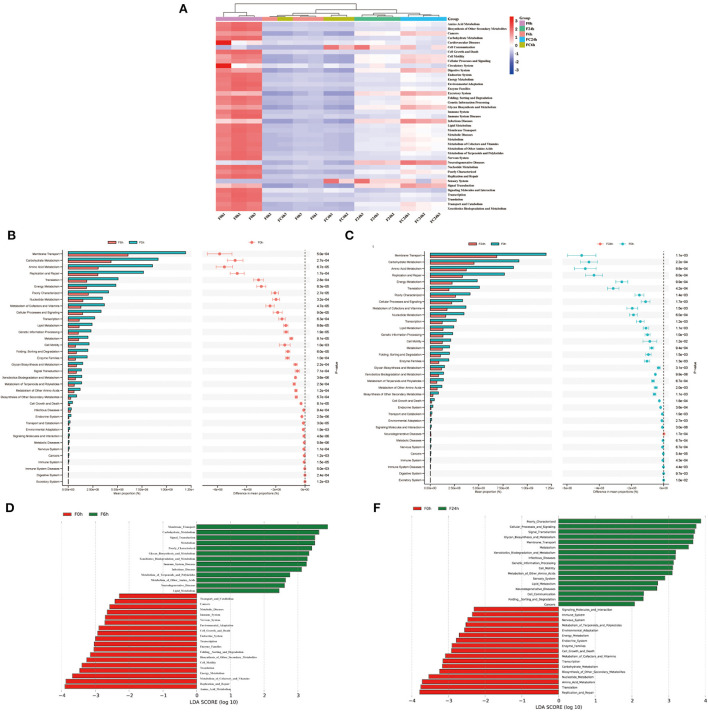
KEGG pathway analysis. **(A)** The heatmap of clustering for KEGG pathways. **(B,C)** Analysis for KEGG pathway using STAMP of F6h and F24h compared to F0h. **(D,E)** Analysis for KEGG pathway using Lefse of F6h and F24h compared to F0h.

Genes and Genome (KEGG) analysis between F0 h, F6 h, F24 h, FC6 h, FC24 h is shown in Figure 10A. Over all, the most functions were significantly decreased after colonic fermentation (*p* < 0.05), but F24 h was still significantly greater than the F6 h. Surprisingly, in the F24 h groups, the metabolic pathways, such as infectious diseases, metabolic diseases and neurodegenerative diseases, decreased compared with that of the FC24 h. From Figures 10B,D, F6 h was primarily mediated by membrane transport, carbohydrate metabolism, amino acid metabolism, replication and repair and translation. From Figures 10C,E, F24 h was primarily mediated by membrane transport, carbohydrate metabolism, amino acid metabolism, replication and repair and energy metabolism. Among them, amino acid metabolism has been shown to be related to SCFAs generation (44). This also explains the contents of SCFAs significantly increased after fermentation. Additionally, compared with F0 h, during the fermentation, the metabolism related to diseases gradually decreased to a lower level. Therefore, the above research results show that FJR may have the potential function of protecting the intestinal in this model.

## Discussion

FJR has good nutritive value as well as good functional properties, the active compound and bioactivities changes during the digestion process have been poorly studied, which hindered further utilization of its nutritive and medicinal value.

As a result, the TPC and TFC of FJR were released most in the oral digestion, which was much higher than gastric and intestine. Meanwhile, these changes effected in release of compounds with antioxidant abilities (DPPH, ABTS and FRAP), α-glucosidase and α-amylase inhibitory abilities. In summary, the bioactivities of FJR were affected by the *in vitro* gastrointestinal digestion and colonic fermentation, which in turn led to significant decreasing. In comparison to the undigested sample, it still maintained certain antioxidant and α-glucosidase and α-amylase inhibitory abilities *in vitro*.

Moreover, 13 phenolic acids and 3 flavonoids of FJR were identified. In general, there was a falling trend in compounds released during fermentation. However, salicylic acid, protocatechuic acid, ellagic acid, vanillic acid, p-coumaric acid and caffeic acid were released increased during the oral phase and intestine phase, respectively. Of which, caffeic acid was only detected in the intestine phase. These data indicate that the part of these compounds in the fermentation supernatants likely came from microbial transformation.

Additionally, after fermentation for 24 h, FJR promoted the production of SCFAs, and the concentrations were significantly higher than their original levels (p <0.05) after 24 h colonic fermentation. Then FJR could decrease the pH of the contents of colonic fermentation, which was favorable for improving the activity of intestinal digestive enzymes, promoting digestion and absorption, and maintaining intestinal environmental stability in colonic fermentation. The results of the 16 S rRNA gene sequence analysis also showed that the microbe community structure was regulated by fecal fermented FJR through improving OTU of beneficial microbiota and suppressing metabolism related to diseases.

## Conclusions

In summary, this study evaluated the dynamic change of the active components and functional activities of FJR during the *in vitro* gastrointestinal digestion and colonic fermentation, and explored the effects of FJR on fecal microbial diversity. The results showed that bioactivities and bioaccessibility of FJR were significantly affected by the *in vitro* gastrointestinal digestion and colonic fermentation. Additionally, pH decreased during the fermentation and SCFAs contents considerably increased. Our results reveal that FJR could improve intestinal microbial community richness, and suppress metabolism related to diseases. Therefore, FJR is expected to be a functional food of antioxidant, hypoglycemic, and promoting gut health. Overall, the present study provides a reference for the development and utilization of FJR. However, it is necessary to further verify theirs *in vivo* digestive properties and mechanism of action in future studies.

## Data availability statement

The datasets presented in this study can be found in online repositories. The names of the repository/repositories and accession number(s) can be found below: BioProject, accession number PRJNA868134.

## Author contributions

ZW and YR: methodology. ZW and XJ: validation. XZ: formal analysis. YR: data curation. XJ: writing—original draft preparation. ZW and XJ: writing—review and editing. PX and XH: supervision. XZ and JS: project administration. XH and HK: funding acquisition. All authors have read and agreed to the published version of the manuscript.

## Funding

This work was supported by Yunnan Agricultural Joint Special General Project (202101BD070001-109 and 202101BD070001-089), Yunnan Special General Projects of Basic Research (202201AT070049), China Agriculture Research System of MOF and MARA (CARS-21), Major Science and Technology Project of Yunnan and Kunming (202102AE090042, 202204BI090003, 202205AF150018, and 2021JH002), National and Provincial Excellent Scientist Supporting Programme, and Yunnan Zhengwenjie Expert Workstation (202205AF150018).

## Conflict of interest

The authors declare that the research was conducted in the absence of any commercial or financial relationships that could be construed as a potential conflict of interest.

## Publisher's note

All claims expressed in this article are solely those of the authors and do not necessarily represent those of their affiliated organizations, or those of the publisher, the editors and the reviewers. Any product that may be evaluated in this article, or claim that may be made by its manufacturer, is not guaranteed or endorsed by the publisher.
